# When Delusion Becomes Reality: A Six‐Year Diagnostic Odyssey and Ethical Crisis in a Case of Veridical Spousal Infidelity Belief

**DOI:** 10.1155/crps/3752206

**Published:** 2026-04-27

**Authors:** Alireza Parsapoor, Mehdi Sayyah

**Affiliations:** ^1^ Medical Ethics and History of Medicine Research Center, Tehran University of Medical Sciences, Tehran, Iran, tums.ac.ir; ^2^ Education Development Center, Jundishapur University of Medical Sciences, Ahvaz, Iran, ajums.ac.ir

**Keywords:** confidentiality, Delusional Disorder, diagnostic error, epistemic injustice, medical ethics, Othello syndrome, treatment resistance, veridical delusion

## Abstract

**Background:**

The diagnosis of delusional disorder, jealousy type, traditionally relies on the criterion of a “false” belief. This report describes a complex case in which a belief long considered delusional was ultimately verified, revealing limitations of rigid diagnostic frameworks and raising a profound ethical dilemma.

**Case Presentation:**

Ms. A, a 42‐year‐old woman, presented with a fixed, 4‐year belief in her husband’s infidelity, accompanied by mild depressive symptoms but preserved global functioning. Over a 6‐year period, she received treatment from multiple psychiatrists, including sequential trials of antipsychotics (risperidone, olanzapine, and aripiprazole) without benefit. Documented treatment resistance led to a trial of electroconvulsive therapy (ECT). Following the first ECT session, her husband confessed to long‐term infidelity and a secret remarriage, while demanding that the clinician withhold this information from the patient.

**Discussion:**

This case challenges the diagnostic emphasis on belief falsity and illustrates how prolonged familial deception can sustain misdiagnosis. The protracted, multiprovider treatment history likely contributed to diagnostic entrenchment and therapeutic escalation. Ethically, maintaining secrecy after the confession constituted institutional gaslighting and epistemic injustice, violating core principles of nonmaleficence and autonomy. The clinician’s primary duty to the patient must supersede spousal demands for confidentiality in cases of harmful deception.

**Conclusion:**

Veridical “delusions” require a paradigm shift toward dynamic diagnosis and patient‐centered ethics. Clinicians must critically evaluate collateral information, remain alert to epistemic injustice, and prioritize patient welfare and truth disclosure over preservation of harmful familial secrets.

## 1. Introduction

Delusional disorder, jealousy type (commonly known as Othello syndrome), is defined in DSM‐5‐TR as the presence of a nonbizarre delusion involving a partner’s infidelity, lasting at least 1 month, without prominent other psychotic symptoms or underlying medical conditions [1]. Although the criterion of a “false” belief is central to this definition, determining truth in intimate relationships often depends on incomplete or misleading collateral histories [2]. Philosophers and psychiatrists have long questioned the necessity of falsity; authors such as Spitzer [3] and Coltheart [4] argue that the essence of a delusion lies in its unjustified, fixed, and incorrigible nature rather than its objective truth value.

We present a case that vividly illustrates this diagnostic and ethical challenge: a woman treated for delusional jealousy over 6 years by multiple clinicians, who ultimately proved correct in her belief. This report details her diagnostic and therapeutic odyssey, analyzes factors contributing to diagnostic entrenchment and treatment escalation, and examines the ethical conflict that arose when the assumption of falsity collapsed. We argue for a more nuanced, dynamic diagnostic approach and an unwavering commitment to patient autonomy and protection from iatrogenic harm.

## 2. Case Presentation

### 2.1. Initial Presentation and Clinical Course

Ms. A, a 42‐year‐old married woman and mother of two with no prior psychiatric history, presented with a 4‐year history of an unshakable conviction that her husband was unfaithful. She described gathering “clues” such as perceived changes in routine and unaccounted expenses, which she regarded as incontrovertible evidence. Associated symptoms included moderate dysphoria, anxious preoccupation, and insomnia. Mental status examination showed no formal thought disorder, hallucinations, mood‐congruent psychotic features, or cognitive impairment. Her socio‐occupational functioning remained intact. A provisional diagnosis of delusional disorder, jealousy type was made, based on the apparent falsity of the belief, which was reinforced by the husband’s consistent denials during collateral interviews.

### 2.2. Longitudinal Treatment History and Escalation

Over the following 6 years, Ms. A was managed by several psychiatrists in both private and tertiary settings. Treatment remained predominantly biological:

• Pharmacotherapy: Sequential adequate trials of risperidone (up to 4 mg/day for 6 months), olanzapine (up to 15 mg/day for 8 months), and aripiprazole (up to 20 mg/day for 9 months) produced no meaningful reduction in the intensity or fixity of her belief or associated distress.
• Electroconvulsive therapy (ECT): Given documented treatment resistance and reported (though not formally recorded) familial pressure, bitemporal ECT was proposed as a last resort for “severe, treatment‐refractory delusional disorder causing significant distress.” Although ECT is generally reserved for mood disorders or catatonia, its use in treatment‐refractory delusional disorder has been reported, though with known cognitive risks [5].

### 2.3. The Pivotal Revelation and Ethical Confrontation

Immediately after the first ECT session, the husband requested an urgent private meeting with the treating psychiatrist. He confessed to a concurrent romantic relationship lasting over 5 years and a secret second marriage 2 years earlier, stating, “She was right all along. I’ve been lying to everyone.” He demanded immediate cessation of ECT and absolute secrecy from his wife, warning of catastrophic family consequences (divorce or suicide) and hinting at potential legal action against the clinician.

### 2.4. The Immediate Clinical Impasse

The confession invalidated the core diagnostic assumption: the belief was not false. The psychiatrist now faced a dilemma—continuing ECT to “treat” a veridical belief (thus participating in gaslighting) or halting treatment while deciding whether and how to disclose information conveyed under an expectation of confidentiality.

## 3. Discussion

### 3.1. Diagnostic Rigidity and the “Falsity” Criterion: A Critical Reappraisal

This case highlights the limitations of rigidly applying the “falsity” criterion in DSM‐5‐TR. For 6 years, the husband’s consistent narrative and the patient’s lack of “hard” evidence were interpreted as confirmation of delusion. The patient’s unyielding conviction across multiple clinicians was viewed as pathological rigidity rather than a possibly accurate perception [6]. This aligns with critiques emphasizing that delusionality resides in abnormal belief formation and maintenance, not necessarily in objective falsity [4].

#### 3.1.1. Jaspers’ Phenomenological

Approach to delusion and veridical content according to Jaspers, delusions may not be objectively false insofar as the content is concerned. This is best exemplified in delusional jealousy, whereby the belief may correspond to objective truth and is therefore not false. Delusions may not be held with extraordinary conviction, but, equally, normal beliefs may be held with extraordinary conviction. Delusional beliefs may also be amenable to counterargument, although it is rare that this by itself will alter the belief. Finally, delusional content need not be impossible [7].

Applying these criteria to the present case, several features support the diagnosis of delusion despite the possibility of veridical content: fixed, unshakeable conviction persisting over 6 years; systematic overinterpretation of minor “clues” (e.g., expenses, changes in routine, ambiguous behaviors); significant distress manifested as insomnia and anxiety; and a belief that appeared disproportionate to the initially available evidence. The truth of the belief does not automatically rule out a delusion—but it makes the diagnosis much harder and requires clear evidence of pathological reasoning. In this case, the patient’s conviction regarding expenses and routine changes represented a clear overinterpretation of minor clues, fulfilling the criterion of abnormal belief formation even if parts of the final conclusion later proved veridical [3].

### 3.2. Unpacking the Trajectory Toward Aggressive Intervention

The decision to proceed with ECT emerged from a 6‐year narrative of treatment failure. Each successive clinician encountered what appeared to be classic treatment‐resistant delusional disorder. The preservation of social functioning may have paradoxically encouraged escalation, as eliminating the single “encapsulated” false belief seemed sufficient to restore well‐being. Multiple treaters likely contributed to diffusion of responsibility and reduced incentive for diagnostic re‐evaluation. Unrecorded familial pressure from the husband further tilted the clinical environment toward more invasive interventions, risking psychiatry’s unwitting complicity in perpetuating deception.

### 3.3. Ethical Analysis: A Hierarchy of Duties in the Face of Deception

The ethical conflict reveals a clear hierarchy of principles [8]:

• Nonmaleficence: Continuing treatment based on a false premise, or withholding the truth after confession, constituted active participation in institutional gaslighting and inflicted chronic psychological harm.
• Autonomy and epistemic injustice: Ms. A was deprived of her status as a credible knower—a clear example of testimonial injustice [9, 10]. Informed autonomy is impossible in orchestrated ignorance.
• Confidentiality and its limits: The husband’s disclosure was not a therapeutic confession but a strategic attempt to maintain control. Ethical codes [11] permit breaching confidentiality when secrecy itself causes serious harm to the identified patient. The clinician’s primary fiduciary duty remains with Ms. A.

### 3.4. Cultural Context and Universal Ethics

In collectivist societies such as Iran, strong emphasis on family harmony can exert subtle pressure on clinicians to prioritize familial stability. However, universal biomedical ethical principles must prevail. Cultural relativism cannot justify prolonging deception that inflicts direct harm on an individual patient.

## 4. Conclusion

Ms. A’s 6‐year journey underscores the vulnerability of psychiatric diagnosis to manipulation and the ethical risks of rigid adherence to the falsity criterion. Key lessons include:

1. Adoption of dynamic, hypothesis‐testing diagnostic models open to revision upon new evidence.
2. Greater scrutiny of collateral information, especially in jealousy cases, while guarding against epistemic injustice.
3. Integration of concepts such as epistemic injustice and gaslighting into psychiatric training.
4. Development of institutional protocols to support clinicians facing conflicts between patient welfare and familial demands for secrecy.

When a presumed delusion is revealed as reality, the clinician’s role must shift from treating nonexistent pathology to advocating for the patient’s right to truth and supporting her through its consequences. Patient welfare and nonmaleficence must always take precedence over preserving a harmful lie.

## Funding

No funding was received for this manuscript.

## Conflicts of Interest

The authors declare no conflicts of interest.

## Data Availability

The data that support the findings of this study are available upon request from the corresponding author. The data are not publicly available due to privacy or ethical restrictions.

## References

[1] American Psychiatric Association , Diagnostic and Statistical Manual of Mental Disorders, 2022, American Psychiatric Association, (5th ed., Text Revision).

[2] Grover S. , et al.Delusional Disorder With Jealousy: A Case Series, Indian Journal of Psychiatry. (2010) 52, no. 3, 260–263.21180413

[3] Spitzer M. , On Defining Delusions, Comprehensive Psychiatry. (1990) 31, no. 5, 377–397, 10.1016/0010-440X(90)90023-L, 2-s2.0-0025024064. 2225797

[4] Coltheart M. , Cognitive Neuropsychiatry and Delusional Belief, Quarterly Journal of Experimental Psychology. (2007) 60, no. 8, 1041–1062, 10.1080/17470210701338071, 2-s2.0-34547533177. 17654390

[5] Semkovska M. and McLoughlin D. M. , Objective Cognitive Performance Associated With Electroconvulsive Therapy for Depression: A Systematic Review and Meta-Analysis, Psychological Medicine. (2010) 40, no. 12, 1973–1985. 10.1016/j.biopsych.2010.06.009 20673880

[6] Munro A. , Delusional Disorder: Paranoia and Related Illnesses, 1999, Cambridge University Press.

[7] Sims A. , Symptoms in the Mind: An Introduction to Descriptive Psychopathology, 2003, 3rd edition, W.B. Saunders.

[8] Beauchamp T. L. and Childress J. F. , Principles of Biomedical Ethics, 2019, 8th edition, Oxford University Press.

[9] Fricker M. , Epistemic Injustice: Power and the Ethics of Knowing, 2007, Oxford University Press.

[10] Sanati A. and Kyratsous M. , Epistemic Injustice in Assessment of Delusions, Journal of Evaluation in Clinical Practice. (2015) 21, no. 3, 479–485, 10.1111/jep.12347, 2-s2.0-84928823851. 25828924

[11] Medical Council of the Islamic Republic of Iran , *Code of Professional Ethics* (Persian), 1999.

